# Decision-making conversations for life-sustaining treatment with seriously ill patients using a Danish version of the US POLST: a qualitative study of patient and physician experiences

**DOI:** 10.1080/02813432.2022.2036481

**Published:** 2022-02-11

**Authors:** Lone Doris Tuesen, Anne Sophie Ågård, Hans-Henrik Bülow, Erik K. Fromme, Hanne Irene Jensen

**Affiliations:** a Department of Anaesthesiology and Intensive Care, Vejle and Middelfart Hospitals, University Hospital of Southern Denmark, Vejle, Denmark; b Department of Regional Health Research, University of Southern Denmark, Odense, Denmark; c Department of Intensive Care, Aarhus University Hospital, Aarhus, Denmark; d Department of Public Health-Nursing, Aarhus University, Aarhus, Denmark; e Department of Anaesthesiology and Intensive Care, Holbaek Hospital, Holbaek, Denmark; f Ariadne Labs, A Joint Center for Health Systems Innovation at Brigham and Women’s Hospital and Harvard T.H. Chan School of Public Health, Boston, MA, USA

**Keywords:** Shared decision-making, life-sustaining treatment, end-of-life, interviews, qualitative study

## Abstract

**Objective:**

To explore patients’ and physicians’ perspectives on a decision-making conversation for life-sustaining treatment, based on the Danish model of the American Physician Orders for Life Sustaining Treatment (POLST) form.

**Design:**

Semi-structured interviews following a conversation about preferences for life-sustaining treatment.

**Setting:**

Danish hospitals, nursing homes, and general practitioners’ clinics.

**Subjects:**

Patients and physicians.

**Main outcome measures:**

Qualitative analyses of interview data.

**Findings:**

After participating in a conversation about life-sustaining treatment using the Danish POLST form, a total of six patients and five physicians representing different settings and age groups participated in an interview about their experience of the process. Within the main research questions, six subthemes were identified: Timing, relatives are key persons, clarifying treatment preferences, documentation across settings, strengthening patient autonomy, and structure influences conversations. Most patients and physicians found having a conversation about levels of life-sustaining treatment valuable but also complicated due to the different levels of knowledge and attending to individual patient needs and medical necessities. Relatives were considered as key persons to ensure the understanding of the treatment trajectory and the ability to advocate for the patient in case of a medical crisis. The majority of participants found that the conversation strengthened patient autonomy.

**Conclusion:**

Patients and physicians found having a conversation about levels of life-sustaining treatment valuable, especially for strengthening patient autonomy. Relatives were considered key persons. The timing of the conversation and securing sufficient knowledge for shared decision-making were the main perceived challenges.

**KEY POINTS**
- Conversations about preferences for life-sustaining treatment are important, but not performed systematically.
- When planning a conversation about preferences for life-sustaining treatment, the timing of the conversation and the inclusion of relatives are key elements.
- Decision-making conversations can help patients feel in charge and less alone, and make it easier for health professionals to provide goal-concordant care.
- Using a model like the Danish POLST form may help to initiate, conduct and structure conversations about preferences for life-sustaining treatment.

## Introduction

Worldwide the number of people with chronic or long-term illnesses has increased and will continue to do so [1]. Advanced Care Planning (ACP) is a process supporting patients and their caregivers in their reflection about the meaning and consequences of current and future medical treatments and caregiving [2]. Conversations about preferences for life-sustaining treatment are perceived as worthwhile by patients, family members, and clinicians [3,4]. Yet despite the positive attitude toward conversations about life-sustaining treatment, these conversations are not performed systematically in hospitals and nursing homes [5,6].

There is a lack of knowledge about who is responsible for initiating these conversations, and when and under what circumstances they should be conducted [7,8]. This may mean that there is little opportunity for patients and their relatives to address end-of-life (EOL) issues, leaving important questions unanswered. Patients may not fully understand their illness, prognosis, and treatment options or may not receive medical care consistent with their values and goals [9].

The decision to limit or discontinue treatment can be a difficult issue, not only for patients but also for physicians and other health care professionals. One ACP model to help ensure that seriously ill patients’ treatment preferences are elicited, communicated, and honoured, is the American POLST (Physician Orders for Life-Sustaining Treatment) form [10]. The POLST form addresses patients’ preferences regarding cardiopulmonary resuscitation (CPR), medical interventions, and artificially administered nutrition. The POLST is not just a form; it is a tool to facilitate shared decision-making, with emphasis on the conversation between the patient and healthcare professionals about the patient’s goals of care considering the current diagnosis, prognosis, and treatment options [10]. Use of the POLST form is always voluntary [11].

Less aggressive EOL care has been observed when health care professionals discuss approaching EOL and preferences about life-sustaining treatments with nursing home residents or their families [12]. However, little is known about how these conversations are experienced by patients and physicians in hospitals and nursing homes [13]. Applying strategies to make patient preferences known to healthcare providers and support persons while the patient still has the capacity for this is a critical step in improving the quality of EOL care [14]. A specific template for conducting such conversations may be an important aid for physicians.

Modelled on the US POLST form, we developed and tested a Danish version of the POLST form to facilitate making patients’ preferences for levels of life-sustaining treatment known and documented. The aim of this study was to explore patients’ and physicians’ perspectives on a decision-making conversation for life-sustaining treatment based on the Danish version.

## Materials and methods

### Design

From August 2017 to July 2020, a Danish version of a POLST form based on three different US POLST forms [10] was developed and tested with patients, relatives, and clinicians from hospital wards, general practitioners’ clinics, and nursing homes. The Danish POLST version consists of three sections on cardiopulmonary resuscitation (CPR), medical interventions, and artificially administered nutrition (Supplementary Material SM 1). The process was evaluated by questionnaires and in-depth interviews.

### Participants and settings

Project sites included hospitals, nursing homes, and general practitioners. All sites were visited by the first author and introduced to the Danish POLST form and the study. After the introduction, staff members at the sites were identified along with eligible patients. The patient inclusion criteria were: (I) 18 years or older; (II) patients with serious illness and/or frailty such that the physician would not be surprised if the patient died within the next 12 months; (III) ability to read and understand Danish; (IV) no known cognitive impairment. The physician and patient had a conversation based on the POLST form. Depending on the patients’ preferences, one or more family members and/or nursing staff could participate. At the end of the conversation, the patient’s preferences were documented. As the POLST form was not yet a legal document, the preferences were also documented in the patient’s medical record. The healthcare professionals did not receive specific training in conducting a decision-making conversation. However, the project material included a list of ‘helpful prompts and questions’ to start, conduct, and complete the conversation. The participants agreed to be contacted for a potential follow-up interview, conducted as an extended part of the validation process to gain a wider understanding of how patients and physicians experience an ACP conversation; in this case, based on the Danish POLST form.

### Interviews

The current study presents results from the follow-up interviews which were conducted as individual interviews according to Kvale’s and Brinkmann’s guidelines [15]. The semi-structured interview guide included four key themes: (1) Preparing for the decision-making conversation. (2) Particular challenges. (3) The most important aspect of the conversation. (4) The usability of the Danish POLST form. The four key themes were generated from the participants’ assessments and comments in a survey questionnaire for all participants and the experiences in a previous POLST pilot study (not yet published). In the interview guide, each key theme was addressed through a research question with several sub-questions guiding the in-depth interviews. The interview guide is available as Supplementary Material (SM 2). All interviews were conducted by the first author over an eight-month period, digitally recorded, and transcribed verbatim.

The purpose of the interviews was to examine how the patients and the physicians experienced the decision-making conversation based on the Danish version of the POLST form. The interviews were conducted in the last part of the inclusion period. To get as many perspectives as possible, we recruited participants from different settings and age groups, and physicians and nurses from the different settings collaborated in selecting which of the patients were in a stage of their illness where it was appropriate to approach them. Participants were invited by email to participate in the interviews. Due to COVID-19, to ensure that all invited participants were able to share their perspectives on the POLST decision-making process, the design of the study was changed from face-to-face interviews to telephone interviews based on participants’ individual choices. One physician interview and all patient interviews were conducted as telephone interviews.

### Ethics

All interviewees were informed orally and in writing about the study and gave written consent. The Committee on Health Research Ethics for Southern Denmark assessed the study and concluded that according to Danish law the study did not require ethical approval (29 March 2017). The study was registered with the Danish Data Protection Agency (1732459). To ensure data security, a license agreement was obtained with OPEN (Open Patient Data Explorative Network) (OP_504). The study was in accordance with the ethical standards of the responsible committee on human experimentation and with the Helsinki Declaration of 1975, as revised in 1983.

### Analysis

We used thematic analysis as described by Braun and Clarke [16] as the analytic strategy. First, the interviews were read several times open-mindedly to gain an overall comprehension of the material while making notes about preliminary analytic ideas. Second, meaning units were identified in the text and coded with preliminary codes. In the third step, we searched for themes by looking at the list of codes and their associated text excerpts, constantly comparing and collating the codes into broader potential themes in accordance with the aim of our study. Fourth, the text extracts were checked to see if they still supported the theme. In the fifth step, the themes were reviewed and refined. If any inconsistencies were detected or themes had become too broad, themes were subdivided or codes were moved into an existing theme where they fit better. We kept reviewing and refining the themes until each theme was coherent and distinct. As part of the refinement, subthemes were identified. The NVivo 12 computer program was used to assist the process of analysis.

## Findings

A total of six patients (from departments of Oncology, Haematology and Respiratory Medicine, a nursing Home, and a General Practitioners Clinic) were invited to participate in a follow-up interview. Patient characteristics are presented in Table 1. Furthermore, five physicians (representing four departments: oncology, haematology, neurology, geriatrics) were invited to participate (four female physicians and one male physician, aged 41–58 years). All invited participants accepted the invitation. The interviewees were invited from the main study group of 95 patients and 28 physicians and selected so that they represented primary and secondary health care, different specialities, and different age groups. Table 1 presents patient characteristics from the interview study group. Supplementary Table S1 (SM 3) presents the patient's characteristics of the main group.

**Table 1.** Characteristics of patient participants.

Sample characteristics	Interview study group (*n* = 6)
Gender, *n* (%)	
Male	3 (50)
Female	3 (50)
Age, years, *n* (%)	
40–64	2 (33)
65–74	1 (17)
75–84	2 (33)
85+	1 (17)
Treatment preferences	
Section A. Cardiopulmonary resuscitation	
Attempt resuscitation	2 (33)
Do not attempt resuscitation	4 (67)
Section B. Medical interventions	
Comfort measures only	0 (0)
Selected treatment	4 (67)
Full treatment	2 (33)
Section C. Artificially administered nutrition	
Do administer artificial nutrition	1 (17)
Do not administer artificial nutrition	5 (83)
Setting, *n* (%)	
Hospital	4 (66)
General practitioner	1 (17)
Nursing home	1 (17)

The patient interview study group was fairly similar to the main study group in terms of gender, age and setting, but with a higher proportion of patients representing the treatment preferences: ‘Attempt resuscitation’ and ‘Full treatment’. One patient agreed to participate in the interview despite having shortness of breath. However, as the patient’s respiratory problems increased, the interview was terminated after 5 min (IP [Interview person] 9). Another patient several times reported a lack of memory, saying ‘*I do not remember*’ (IP8).

The interviews were conducted from November 2019 to September 2020. The interviews lasted 5–75 min (mean of 34 min) and took place between 9 and 25 days after the POLST-conversation apart from one patient interview, which took place 10 months after the POLST-conversation. Within the four key themes, subthemes were identified. The themes apply to both patients and physicians, and an overview of key themes, subthemes, and main content can be found in Table 2.

**Table 2.** Key themes, subthemes, and content of patients’ and physicians’ perspectives on decision-making for life-sustaining treatment.

Themes and subthemes	Patients’ perspectives	Physicians’ perspectives
1. Preparing for the decision-making conversation (key-theme)		
Timing	Different levels of preparedness for the conversations but even so, conversations about treatment options and preferences were desired.	Balance between individual patient consideration and medical necessity. Early conversations much better than later. Conversations dependent on physician initiative.
Relatives as key persons	Relatives’ presence during conversations is important for being aware of patient preferences, and for later on being able to speak on behalf of the patient.	Relatives’ presence supports explicating patient preferences, sheds light on possible conflicts between patient’s and relatives’ opinions, and helps clarify the treatment trajectory.
2. Particular challenges (key-theme)		
Clarifying treatment preferences	Explicating preferences may be difficult due to different levels of knowledge about treatment options and consequences.	Clarifying treatment preferences is complicated due to lack of organisational culture, lack of communication skills, need to address both treatment and non-treatment consequences, and family culture.
Documentation across settings	Patients’ experiences of cross-sectoral documentation ranged from fine to non-existing.	Difficult to secure documentation of preferences across settings due to different IT-systems.
3. The most important aspect of the conversation (key-theme)		
Strengthening patient autonomy	Shared decision-making about life-sustaining treatment makes the patients feel in charge, less alone, empowered, secure that relatives will not have to guess the preferences, and makes it easier for health professionals to provide goal-concordant care	Making patients understand they have a choice, also for opting out. Comprehensive and on-going information about implications of preferences is necessary.
4. Usability of the Danish POLST form (key-theme)		
Structure influences decision-making conversations	The form may help to make the conversation concrete and less committing.	The form is helpful in initiating, structuring, including patient perspectives and defusing the conversation, but may also disturb the conversation.

### Preparing for the decision-making conversation

Two overarching subthemes of preparing for the decision-making conversation were identified: Timing and relatives as key persons.

#### Timing

##### The patients’ perspective

To several of the patients, being invited to a decision-making conversation about preferences for resuscitation and life-sustaining treatment did not come as a surprise:

‘*It has been on my mind all the time, I have been thinking about this (life-sustaining treatment) all the time, that is nothing new.*’ – IP9

These patients expressed that before the decision-making conversation, they had already decided on their preferences for CPR as well as artificial nutrition. However, one patient questioned her choice as she remembered a fellow patient receiving artificial nutrition, indicating that choices may change over time.

A patient faced with a fast-progressing illness was not prepared for the conversation:

‘*It was shocking to me. At first, I could still be cured. Then you are suddenly incurable and then at the next scan, you are suddenly a terminal patient. It all happened very fast, I don’t think you can ever prepare for that.*’ – IP6

Especially younger patients, aged 40–64, expressed the emotional strain in facing progressing illness, and at the same time wishing for longer life, making it difficult to consider anything but to live as long as possible. However, irrespective of the experience with the timing of the POLST decision-making conversation, the patients expressed that they wanted to have the conversation even it could be emotionally difficult.

##### The physicians’ perspective

Several physicians addressed the need to clarify the patients’ preferences for levels of treatment. They found it far too late to do so once the patient was seriously ill and hospitalized. Having the decision-making conversation was therefore also regarded as a collegial task as clarity about patient preferences would also be helpful to other physicians during a medical crisis.

Several physicians experienced that the patients, when invited to participate, often would accept the invitation without hesitation. Verifying if the patient was open to having the conversation or requested more information was a strategy developed by some of the physicians to find the right timing before explaining a difficult diagnosis. Other physicians had a different approach:

‘*The patients are very ill and the illness progresses fast, we cannot wait to have the conversation next time, we must seize the chance when the patient is here.*’ – IP2

Timing means finding the right time for the patient. This was described as knowing the patient’s status of illness and treatment trajectory, which topics would be important to address, and when it was acceptable to have the conversation with the patient. Likewise, having the decision-making conversation early in the patient's course of illness could make some of the conversations less critical and more open, as the preferences would be discussed apart from a medical crisis.

Regarding timing, one physician commented:

‘*I also think that they (the patients) want to talk about all this earlier than we (the physicians) do.*’ – IP3

But despite experiencing that the majority of patients wished to have the conversation, several of the physicians had met patients who declined the decision-making conversation. The physicians found it was too overwhelming for some patients, who responded with confusion or by being dismissive as the conversation became more specific.

The physicians also found that many patients were completely dependent on professionals taking the initiative to talk about EOL issues. When the physician took the initiative, the patients realized there was a need to have the conversation. Finding the right time for the decision-making conversation also depended on making sure that the right people were with the patient during the conversation. The patient's spouse and children were the relatives mentioned by most physicians as the right people to bring in for the conversation.

#### Relatives are key persons

##### The patients’ perspective

Nearly all patients referred to the importance of relatives being present during the decision-making conversation. They expressed that it was important to have the relatives witnessing their preferences, so the relatives would know and be able to describe the preferences accurately on behalf of the patient. This was emphasised even more by one of the patients, who realised her ACP wishes were not documented across the country, as unfortunately, the five Danish regions have different systems for documentation in medical records.

Despite having the relatives present during physician consultations and, for the hospitalised patients, during treatments and examinations, none of the patients had discussed in detail their preferences for life-sustaining treatment with their relatives before the conversation:

‘*We do not really get into it …. but enjoy everyday life as long as we can. We have not made any common path yet, no, we have not reached that.*’ – IP11

##### The physicians’ perspective

The physicians emphasised the importance of relatives being present during the decision-making conversation, using words like ‘crucial’, ‘essential’, and ‘most important’. They also found it important that the Danish POLST form documents if relatives are present or not. Not only did the physicians find the relatives supportive when explicating the patient’s preferences, but a physician also described them as key persons in the patient’s illness trajectory.

‘*People do not just make decisions alone, they make it in the context they live in now and when you are seriously ill, it is usually with the family and the relatives.*’ – IP1

Some physicians decided to postpone the conversation if the right persons for the patient were not present. Other physicians planned a follow-up conversation instead. Having relatives present could also be an occasion to shed light on possible conflicts between the patients’ preferences and the relatives’ opinions.

Some of the physicians had learned that patients used the copy of the POLST form as the basis for having a conversation at home with the family, sharing their preferences for treatments. For the physicians, having the relative's witness decision-making regarding the treatment plan was important for the course of the patient’s treatment trajectory.

### Particular challenges

Clarifying treatment preferences and documentation of the conversation across settings were identified as particular challenges by both the patients and physicians.

#### Clarifying treatment preferences

##### The patients’ perspective

A key topic for all the patients was that they based their knowledge about medical treatment on their experiences from earlier hospitalisations, or on experiences of the illness trajectories of relatives and friends. They referred to being with parents or siblings at the end of their lives without really knowing what kind of treatments were initiated or not, or they described having conversations with fellow patients that seemed very ill without asking about their fellow patient’s treatment. As one patient expressed:

‘*My mom and dad were comatose at the end, but what kind of treatments they received is something I know nothing about, and we never asked about it…. so it is difficult for me to say, what kind of treatment I prefer*’ – IP10

All the patients expressed their choice regarding CPR with a firm conviction whereas their preferences about artificial nutrition were referred to less conclusively. Some of the patients were unsure if their bodies, despite the serious illness, would still need nutrition. If they were to be unable to eat sufficiently for a longer period of time most of the patients would prefer not to have artificial nutrition. Particularly the decision about medical interventions (section B in the Danish version of the POLST form) was emphasised as a shared decision. The patients described their lack of medical insight as a barrier when discussing their preferences for life-sustaining treatment.

##### The physicians’ perspective

The physicians expressed three different views. To some, addressing treatment preferences was already a natural part of their work. Others described having the communication skills but lacked the culture of discussing treatment preferences with their patients, and the last group had the intent and willingness to discuss ACP with their patients but found they lacked the communicative skills to do such.

Several of the physicians considered decisions about preferences for medical interventions to be particularly challenging as these may imply other dimensions of choices too.

‘*We know a lot about our treatments, what we can do, and how we can act, and what will happen if we do this and so on, but we almost never tell what can happen if we do nothing…. It is another dimension of choice, as it is not possible with factual knowledge to help people on their way to make the choice that is existentially best for them.*’ IP1

Most physicians described the complexity of clarifying treatment preferences. Cultural differences were addressed, e.g. when patients came from a family culture where serious illness and death or dying were not addressed. This was contrasted by experiences with other patients talking openly and freely about preferences for treatment.

#### Documentation across settings

##### The patients’ perspectives

As all patients were seriously ill and/or medically frail, they had experiences with transitions in the Danish healthcare system. Some patients described their illness trajectory as one coherent trajectory. To others it was experienced as transgressive, ‘*like being sent into no-mans-land*.’ – IP6

The patients’ experiences of the cross-sectoral documentation in Danish healthcare ranged from fine to non-existing.

##### The physicians’ perspectives

Several physicians addressed the challenge of documenting the effective communication of patient preferences:

‘*I find that POLST is difficult to document in the hospital system… you can write a correspondence, but where does it land? That is an issue, as the IT systems do not connect.*’ – IP5

### The most important aspect of the conversation

**Strengthening patient autonomy** was the most important aspect identified by both patients and physicians.

#### The patients’ perspective

Most patients explicated the experience of feeling in charge and being the one deciding the limits for treatments.

‘*Now it’s me who decides where the line goes. Instead of them (physicians) deciding where the line goes, you feel that you have a say in things.*’ – IP11

The patients talked about the POLST-conversation as one that made the consultation personal, rather than being given the information. To be given the time to ask questions, listen and talk was also described as making the conversation personal. Some of the patients referred to previously feeling all alone after a consultation, whereas the shared decisions made while filling in the POLST form made the patients feel less alone.

To the patients, quality of life and adherence to treatment were linked together. The main concern for the patients was if their preferences of care and treatment were disregarded:

‘*The best part was to get it down in writing for my family, so they understand my preferences and know how to comply. I do not wish to end up as a vegetable.*’ – IP9

The patients felt empowered by the discussions about their serious illness and medical treatment options. Having their decisions count was important. Also, they wanted to help family members to know their preferences in a medical crisis instead of leaving the family members with the anguish of having to guess the patient’s preferences. The patients also thought the POLST conversation was helpful to the health professionals, allowing them to confidently provide goal-concordant care.

#### The physicians’ perspective

For the patients to understand that they have a choice, also to opt-out of treatment, was emphasised as one of the most important topics. After the conversation, often the patients needed further information about CPR, ICU, artificial nutrition, etc. to fully understand the implications of their preferences. As a physician stated:

‘*If all we have talked about is: this is how it goes — CPR, ICU and artificial nutrition. That is what we can offer. Then it is hard to imagine there is another way. How should they know?*’ – IP1

The physicians respected the patient’s autonomy. They acknowledged that the patients’ preferences also could be very emotional and principled, not always rational, making it important to follow up on the conversation and the decisions made.

However, as emphasised by one of the physicians, from a professional perspective the final decisions also have to be medically meaningful. Some of the physicians stated that if specific treatments options were medically meaningless, the patient would not be given the option, to begin with. A physician stated:

‘*They can agree or disagree with me, and we can talk a little more about it, but they cannot choose something I will not give them.*’ – IP1

### Usability of the Danish POLST form

#### Structure influences the decision-making conversations

##### The patients’ perspective

Most patients valued the conversation but did not have specific comments on the form. However, one patient found that using the form made the conversation feel less committing:

‘*Filling out a form with some things you need to consider along the way or here and now. It is not as committing as if you have to sit and talk directly about those things. Because when you fill out the form, it's not just me that matters, it’s the whole system and everything that comes with it. It is not as formal as when it is the doctor who sits and asks how would you ….*’ – IP4

##### The physicians’ perspective

The physicians found the content of the Danish POLST form simple, relevant, and easy to understand. The majority found that using the form as a structure for the conversation eased both the invitation to and execution of the conversation and the decision-making about life-sustaining treatment:

‘*We have done it before* [had the conversation], *but the fact that it is formalised, and it actually is concretised in this way makes you more aware that it is actually quite simple. Many patients afterwards say that it was really nice to have this conversation.*’ – IP7

Using the document may also help defuse the conversation:

‘*I actually also think that the POLST form can be a relief, as a professional, even if you have had hundreds of such conversations, it can sometimes be a little difficult. How do you handle this constructively when people are sad? I actually think it seems very, very professional. I can see these are really intense things. I can see you are sad. There is actually something we can do to make this easier. I have this document that also helps me to do this in a proper way, these difficult thoughts and feelings.*’ – IP1

However, one physician experienced that the form in part limited the conversation and wanted to be able to conduct the conversation without the specific areas.

## Discussion

### Statement of principal findings

When planning a conversation about levels of life-sustaining treatment, the timing of the conversation and the inclusion of relatives were found to be key elements. Both patients and physicians found that clarifying treatment preferences was important but also complicated, as was securing documentation of preferences across sectors. The most important aspect of the conversations was strengthening patient autonomy, and thereby their participation in decisions concerning the end of their life

### Strengths and weaknesses of the study

Strengths of this study include the participation of both patients and physicians and the representation of different healthcare settings. Furthermore, the semi-structured interview design provided nuanced descriptions of the participants’ experiences. Limitations include the limited number of participants. All participants were seriously ill/frail, and due to the small number of participants, we were unable to identify differences in experiences of the conversation related to diagnosis, speciality, age, or gender. This should be examined in further studies. Furthermore, the results could be influenced by information bias, as it may be the most articulate and positive patients and those who had already come to terms with their preferences who agreed to participate in the interviews. The corona pandemic complicated the inclusion of participants in the study, and data saturation may not have been reached.

### Findings in relation to other studies

Most qualitative studies on patients’ perceptions of end-of-life care are on relational care (who takes care) and fewer on informal care and management continuity [17]. Qualitative studies on physicians’ perceptions of communicating about end-of-life are practically non-existing. However, a recent study has shown that especially outside the Western Hemisphere, health care personnel may have difficulties providing a bad prognosis and hence avoid disclosing it [18]. It is also noteworthy that the majority of studies on POLST come from the USA. The current investigation aimed at closing some of these information gaps.

The current study confirms some prior findings. The timing of a conversation about life-sustaining treatment was considered a key element for both patients and physicians. The physicians found the timing of the conversation to require a balance between patient consideration, not depriving the patient of hope, and medical necessity. Most of the patients did not find the invitation to a conversation about life-sustaining treatment surprising, as before the conversation they had decided their preferences for CPR and nutrition. Even so, most patients had not taken initiative themselves to discuss ACP with their physician. This is in line with other studies where although having thoughts about end-of-life care, less than a third had discussed their preferences with a physician [19,20]. Some physicians in this study had prior experiences where patients had declined a decision-making conversation, but in an English study of severely ill COPD patients, 98 of 100 found EOL discussions appropriate [21]. In contrast, more families than patients seem uncomfortable talking about advanced care planning after it has been brought up by their physician [22], but still three out of four will address the subject several times with the patient afterwards [23]. Lack of clarity about the most appropriate timing has been found in other studies [24]. The ‘surprise question’ (would I be surprised if this patient died in the next 12 months) is a poor to modest predictive tool for death [25], but will still be useful for timing a conversation for levels of treatment in case of emergency for seriously ill patients. The physicians in the current study agreed that having a conversation early, at a stable period in the patient’s illness, was much better than at a late stage or during a medical crisis. This is in line with studies showing that conversations about life-sustaining treatments lead to less aggressive EOL care [12,26]. An early conversation provides time for follow-up conversations if needed, and it helps other clinicians to provide goal-concordant care if a medical crisis occurs.

### Meaning of the study: possible mechanisms and implication for clinicians or policy makers

Including relatives in the conversations about life-sustaining treatment preferences was considered essential by both patients and physicians. For patients, it provided assurance that the relatives now knew, and would help ensuring that the preferences would be honoured. The need for relatives to have actual knowledge about the patient’s preferences instead of having to guess when they are asked to speak on behalf of the patient is supported by research showing discordance between surrogate decision-maker goals for care and medical orders and treatments provided to hospitalised, incapacitated older patients [27]. The physicians found that during the conversations, relatives may support explicating patient preferences, and shed light on possible conflicts between the patient’s and the relative’s opinions. By being part of the conversation about the consequences of different treatment options, relatives would also get a better understanding of the treatment trajectory, thereby decreasing the risk of conflicts between clinicians and relatives [28].

Clarifying preferences and sharing the decision-making for levels of life-sustaining treatment between patients and physicians were considered important by both patients and physicians. However, different levels of knowledge about treatment options and the consequences of different choices can complicate shared decision-making [24,29,30]. Some of the patients had prior experiences with serious illness and death within their family but had little knowledge of which treatments had been provided and why. In shared decision-making, the patient is the expert in his/her own life and the clinician is the expert in prognosis, treatments, and consequences [31]. To be able to make decisions about levels of treatment, the patients need knowledge of treatment consequences, but also of the consequences of not treating. Some of the physicians experienced that patients often just accepted treatment if they were not provided with an alternative; either because patients may not be aware that not receiving life-sustaining treatment is an option, or also because they may be scared that saying no to life-sustaining treatment may result in no treatment at all. Making the patients aware of life-sustaining options *vs.* active treatments is paramount in providing the patients a real option of decision-making [32]. Apart from the challenge of providing sufficient information adjusted to the individual patient, the physicians also found that organisational and individual issues and family culture influenced the decision-making process. Some of the physicians found that clarifying the patients’ treatment preferences and making shared decisions were not part of the organisational culture, and it could therefore be difficult to swim against the tide. For others, lack of belief in their own communicative skills was perceived as a barrier for initiating and conducting conversations [24]. In the current study, the physicians were provided with suggestions for ‘prompt’ questions for introducing the subject and conducting the conversations. Most of the physicians found this sufficient, as they also before the study had experience in conducting conversations about life-sustaining treatment, but to ensure that physicians in general and not just the most confident initiate preference conversations, a more structured aid, and training in conversation skills may be warranted [33]. Likewise, patient-family cultures where death and dying were subjects you did not talk about were perceived as barriers for conducting conversations about life-sustaining treatment. To remove this type of barrier, high-quality communication skills could be helpful [24].

Many patients receive treatments in both primary and secondary healthcare, and one of the challenges identified in the study was difficulties securing documentation of preferences and decisions about life-sustaining treatment across settings due to different IT systems [34]. This is a major patient safety issue that needs to be addressed.

## Conclusions

Patients and physicians found having a conversation about levels of life-sustaining treatment valuable, and relatives were considered key persons. The POLST form can ease both the invitation to and execution of conversations and decisions about life-sustaining treatment. The timing of the conversation and securing sufficient knowledge for shared decision-making were the main perceived challenges.

## Supplementary Material

Supplemental Material

Supplemental Material

Supplemental Material

Supplemental Material
