# Supplementary material for: Decision-making conversations for life-sustaining treatment with seriously ill patients using a Danish version of the US POLST: a qualitative study of patient and physician experiences
Source: Scand J Prim Health Care. 2022 Feb 11;40(1):57–66. doi: 10.1080/02813432.2022.2036481 (PMC9090401; doi:10.1080/02813432.2022.2036481)
Supplement: Supplemental Material [file IPRI_A_2036481_SM7417.docx]

**SM 3.**

**Table S1. Characteristics of patient participants**

| Sample characteristics | Main study group  (n=95) | Interview study group  (n=6) |
| --- | --- | --- |
| Gender. n (%)  Male  Female | 40 (42)  55 (58) | 3 (50)  3 (50) |
| Age, years. n (%)  40-64  65-74  75-84  85+ | 15 (16)  19 (20)  35 (37)  26 (27) | 2 (33)  1 (17)  2 (33)  1 (17) |
| Treatment preferences  Section A. Cardiopulmonary resuscitation  Attempt resuscitation  Do not attempt resuscitation  Section B. Medical interventions  Comfort measures only  Selected treatment  Full treatment  Section C. Artificially administered nutrition  Do administer artificial nutrition  Do Not administer artificial nutrition | 11 (12)  84 (88)  21 (22)  58 (61)  16 (17)  25 (26)  70 (74) | 2 (33)  4 (67)  0 (0)  4 (67)  2 (33)  1 (17)  5 (83) |
| Setting. n (%)  Hospital  General practitioner  Nursing Home | 62 (65)  16 (17)  17 (18) | 4 (66)  1 (17)  1 (17) |
