# Supplementary material for: Decision-making conversations for life-sustaining treatment with seriously ill patients using a Danish version of the US POLST: a qualitative study of patient and physician experiences
Source: Scand J Prim Health Care. 2022 Feb 11;40(1):57–66. doi: 10.1080/02813432.2022.2036481 (PMC9090401; doi:10.1080/02813432.2022.2036481)
Supplement: Supplemental Material [file IPRI_A_2036481_SM7395.docx]

**Projektdokumentet er IKKE juridisk bindende. Beslutninger skal journalføres for at være gyldige**

| **P**atient- **O**g **L**ægebe**s**lutninger for den **S**idste **T**id | | | | 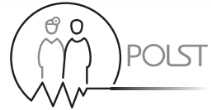 |
| --- | --- | --- | --- | --- |
| Patientens efternavn: | | Patientens for- og mellemnavn: | Patientens cpr.nr.: | |
| Adresse: | | Postnr.: /By: | Dato for udfyldelse: | |
| Evt. pårørende med til samtalen.  Navn: Relation til patient: | | | | |
| **A**  Sæt ét X | **Hjertestop** | | | |
|  | **☐ Forsøg genoplivning.**  Hvis det behandlingsmæssigt giver mening.  **☐ Ingen forsøg på genoplivning.**  Tillad naturlig død. | | | |
| **B**  Sæt ét X | **Lægebehandling** | | | |
|  | **☐ Lindrende behandling – vigtigste mål er mest mulig lindring med behandling af symptomer.**  Lindring af smerte og lidelse. Ønsker ikke indlæggelse på sygehus til livsforlængende behandling. Hvis behov for lindrende behandling ikke kan imødekommes på aktuelle sted, da evt. indlæggelse på sygehus.  **☐ Udvalgt behandling – vigtigste mål er grundlæggende lægebehandling af sygdomstilstand.**  Ud over lindrende behandling gives behandling af sygdomstilstand som f.eks. antibiotika, væske, blodtransfusion og støtte af vejrtrækning uden brug af respirator. Indlæggelse på sygehus ved behov. Undgå som udgangspunkt indlæggelse på intensivt afsnit.  **☐ Fuld behandling – vigtigste mål er livsforlængende behandling.**  Ud over lindrende behandling gives behandling efter behov, herunder indlæggelse på sygehus samt eventuelt intensivt afsnit og respirator behandling, hvis det behandlingsmæssigt giver mening. | | | |
| **C**  Sæt ét X | **Ernæring** | | | |
|  | **☐** **Hvis ikke i stand til at spise tilstrækkeligt i længere tid, ønskes ernæring i sonde eller drop.**  **☐** **Hvis ikke i stand til at spise tilstrækkeligt i længere tid, ønskes *ikke* ernæring i sonde eller drop.** | | | |
| **D** | **Dokumentation af samtale** | | | |
|  | **Underskrift patient**  Dette er mine ønsker, som de er nu. Jeg forstår, at udfyldelse af dette dokument er frivilligt, og at jeg til enhver tid kan ændre mine ønsker.  **Dato:________________ Underskrift:_____________________________________________**  **Underskrift læge**  Patienten er habil. Ovenstående er i overensstemmelse med patientens nuværende sygdomstilstand og ønsker.  **Lægens navn** (blokbogstaver): **Arbejdssted:**  **­­____________________________________ _____________________________________**  **Dato:_______________ Underskrift:______________________________________________** | | | |

| **Evt. kommentarer og ønsker** |
| --- |
|  |
| **Udfyldelse af projektdokument. Vejledning for patienter** |
| - Udfyldelse af projektdokument er **altid frivilligt.** Dokumentet er relevant for dig, hvis du har en kendt sygdom, som f.eks. kræft, KOL eller hjertesygdom, i alvor grad, eller hvis du er generelt svækket pga. alder. Det handler om, at du på forhånd får taget stilling til, hvad du ønsker, at der skal ske, hvis du f.eks. bliver bevidstløs eller får hjertestop. - Dokumentet udfyldes efter en samtale mellem dig (og evt. dine pårørende), din praktiserende læge eller sygehuslæge og evt. plejepersonale. Du kan forinden have gennemgået dokumentet med plejepersonale. I taler om din tilstand og sygdomme og hvilke muligheder, der er for behandling, hvis der sker en forværring. Afhængig af din sygdomstilstand kan der være forskellige behandlinger, som f.eks. respirator, der ikke vil være mulige. - Sammen når I frem til en beslutning om, hvad der er vigtigt for dig, og hvilke ønsker, du har for behandling af sygdom og evt. hjertestop. - Dokumentet registrerer dine ønsker - *som de er nu* - for hjertestop og for behandling. Du kan til enhver tid ændre dine ønsker. |
| **Udfyldelse af projektdokumentet**. **Vejledning for læger og sygeplejersker** |
| - Det er altid frivilligt for patienten at udfylde et projektdokument. - Målgruppen for POLST er patienter med en kendt sygdom, som f.eks. kræft, KOL eller hjertesygdom, i alvor grad, eller patienter, der er generelt svækket pga. alder. - Dokumentet kan gennemgås med patienten af læge eller plejepersonale. Dokumentet skal være underskrevet af både patient og læge. |
| **Gyldighed af beslutninger** |
| - Projektdokumentet er IKKE juridisk bindende. De beslutninger, som patienten tager i samråd med lægen om ønsker for behandling og eventuel genoplivning, skal journalføres efter det enkelte arbejdssteds retningslinjer. - Patienten kan til enhver tid ændre sine ønsker, og disse ændringer journalføres. |
