# Supplementary material for: Decision-making conversations for life-sustaining treatment with seriously ill patients using a Danish version of the US POLST: a qualitative study of patient and physician experiences
Source: Scand J Prim Health Care. 2022 Feb 11;40(1):57–66. doi: 10.1080/02813432.2022.2036481 (PMC9090401; doi:10.1080/02813432.2022.2036481)
Supplement: Supplemental Material [file IPRI_A_2036481_SM7393.docx]

**SM 2. Interview guide. Patients (similar for physicians)**

| Research questions | Aspect of the interview process |  | Interview questions |
| --- | --- | --- | --- |
|  | Intro question | 1 | Do you remember what made you participate in the (POLST) project? |
| What preparations are considered as important prior to the interview? | Transition questions | 2 | How did you prepare for the POLST conversation? |
|  |  | 3 | Did you need to think about anything in particular before you were ready to participate in the POLST conversation? |
|  |  | 4 | Would you have preferred to have seen the POLST document before the conversation? |
|  |  | 5 | What is a good location for the POLST conversation? |
| What are the special challenges of the POLST conversation? | Primary questions | 6 | How were you invited to the POLST conversation? |
|  |  | 7 | Did you consider not participating? (Maybe you can elaborate a little more on this?) |
|  |  | 8 | What is your experience with relatives participating in the POLST conversation? |
|  |  | 9 | What has been the best part of the POLST conversation? |
|  |  | 10 | What has been the hardest part of the POLST conversation?  (The conversation itself or after the conversation) |
|  |  | 11 | Were you surprised by anything? |
|  |  | 12 | Do you have any suggestions that could improve the POLST conversation? |
|  |  | 13 | What do you think about the title of the POLST form…. Patient-and-Physician Decisions for End-of-Life |
|  |  | 14 | Have you changed your current wishes for the level of medical treatment |
| Which "topics" in the POLST conversation are the most important for the patient? |  | 15 | What do you consider most important for your doctor to understand about your wishes for level of medical treatment?  What do you consider most important for your relatives to understand about your wishes for level of medical treatment?  What do you consider most important for the nurses to understand about your wishes for level of medical treatment? |
|  |  | 16 | Is there anything else important that you wish you had talked about? |
|  |  | 17 | What has it given you to participate in the POLST conversation? |
|  |  | 18 | Do you have other comments or ideas you would also like to share? |
